# Sense of coherence and social support as predictors of mental health during COVID-19 pandemic

**DOI:** 10.1590/0034-7167-2022-0468

**Published:** 2023-08-07

**Authors:** Fabiane Dolphine Fuentes Penachiotti, Mirian Ueda Yamaguchi, Adi Mana, Shifra Sagy, Rute Grossi-Milani

**Affiliations:** I Universidade Cesumar. Maringá, Paraná, Brazil; II Cesumar Institute of Science, Technology and Innovation. Maringá, Paraná, Brazil; III Peres Academic Center. Rehovot, Israel; IV Ben-Gurion University of the Negev. Beer Sheva, Israel

**Keywords:** Mental Health, Anxiety, Sense of Coherence, Health Promotion, COVID-19., Saúde Mental, Ansiedade, Senso de Coerência, Promoção da Saúde, COVID-19., Salud Mental, Ansiedad, Sentido de Coherencia, Promoción de la Salud, COVID-19.

## Abstract

**Objectives:**

to verify the role of sense of coherence, sense of national coherence, social support and trust in institutions to predict mental health in Brazilians during the COVID-19 pandemic.

**Methods:**

a cross-sectional study, carried out from November, 2020 to January, 2021 via an online survey. Standardized instruments were used. The sample consisted of 1,630 Brazilians. Pearson’s correlation and linear regression were performed in data analysis.

**Results:**

sense of coherence was the only predictor of anxiety [β= -0.61; p<0.001], explaining 38% of the variance in its scores, while sense of coherence [β= 0.52; p<0.001], sense of national coherence [β= 0.16; p<0.001], and social support [β= 0.15; p<0.001] predicted positive mental health and together explained 51% of its variance.

**Conclusions:**

the findings suggest that sense of coherence, sense of national coherence and social support represent important predictors for mental health and that strengthening these resources, could potentially promote Brazilians’ mental health.

## RESUMO

**Objetivos:**

verificar o papel do senso de coerência, senso de coerência nacional, apoio social e confiança em instituições como preditores da saúde mental dos brasileiros durante a pandemia de COVID-19.

**Métodos:**

estudo transversal, realizado de novembro de 2020 a janeiro de 2021 por meio de questionário online. Foram utilizados instrumentos padronizados. A amostra foi composta por 1.630 brasileiros. Foram realizadas correlação de Pearson e regressão linear na análise dos dados.

**Resultados:**

o senso de coerência foi o único preditor de ansiedade [β=-0,61; p<0,001], explicando 38% da variação em seus escores, enquanto senso de coerência [β=0,52;p<0,001], senso de coerência nacional [β=0,16;p<0,001] e apoio social [β=0,15;p<0,001] foram preditores da saúde mental positiva e juntos explicaram 51% da sua variação.

**Conclusões:**

os achados sugerem que senso de coerência, senso de coerência nacional e apoio social representam importantes preditores da saúde mental e que o fortalecimento desses recursos, poderia potencialmente promover a saúde mental dos brasileiros.

## RESUMEN

**Objetivos:**

verificar el papel del sentido de coherencia, sentido de coherencia nacional, apoyo social y confianza en las instituciones como predictores de salud mental en brasileños durante la pandemia de COVID-19.

**Métodos:**

estudio transversal, realizado de noviembre de 2020 a enero de 2021 mediante cuestionario online. Se utilizaron instrumentos estandarizados. La muestra estuvo compuesta por 1.630 brasileños. En el análisis de datos se realizaron correlación de Pearson y regresión lineal.

**Resultados:**

el sentido de coherencia fue el único predictor de ansiedad [β= -0,61;p<0,001], explicando el 38% de la variación de sus puntuaciones, mientras que el sentido de coherencia [β=0,52;p<0,001], sentido de coherencia nacional [β= 0,16;p<0,001] y apoyo social [β=0,15;p<0,001] fueron predictores de salud mental positiva y en conjunto explicaron el 51% de su variación.

**Conclusiones:**

los hallazgos sugieren que sentido de coherencia, sentido de coherencia nacional y el apoyo social representan importantes predictores de salud mental y que el fortalecimiento de estos recursos, podría potencialmente promover la salud mental de los brasileños.

## INTRODUCTION

The COVID-19 pandemic caused by SARS-CoV-2 spread around the world and created stress and chaos. In addition to disease-related morbidity and mortality, the pandemic impacted people’s mental health and well-being, accounting for a significant increase in mental disorders. In this context, Brazil stood out negatively. Brazilians had their anxiety symptoms worsened^(1)^, and the country that previously had the highest prevalence of anxiety in the world^(2)^ unfortunately remained at the forefront of the disorder during the crisis^(3)^.

Generalized anxiety can be understood as a pathogenic measure, whose symptoms are potentiated by stressful situations^(4)^. On the other hand, mental health is a positive concept related to general coping resources. According the World Health Organization (WHO), the definition of mental health is “a state of well-being in which the individual realizes his or her abilities, can cope with the normal stresses of life, can work productively and fruitfully, and is able to make a contribution to his or her community”^(5)^. And these positive aspects of mental health are contemplated by salutogenesis, which seeks to understand the assets that move people towards having better health and quality of life^(6)^.

Therefore, the current study assessed mental health from two perspectives, positive mental health and generalized anxiety disorder. However, unlike most studies that were focused on the globally negative impact of the pandemic and risk factors associated with mental disorders^(7)^, the current study explored the salutary factors. Employing a salutogenic approach, which seeks to understand the origins of health and health assets, the following question emerged: what are the coping resources and socio-demographic factors related to better mental health and lower levels of anxiety among adults living in Brazil during the COVID-19 pandemic?

This study is part of the “Corona Research Collaboration”, an international project in which researchers from several countries explore the role of personal, social and national coping resources in predicting mental health during the COVID-19 crises.

### Theoretical framework

#### Personal coping resource: sense of coherence

Sense of coherence (SOC) is the main construct of the salutogenic model, which implies the perception of life as comprehensible, manageable, and meaningful^(8)^. According to Antonovsky^(8)^, people with a strong SOC are better able to deal with stressors because they believe they understand the stressor (comprehensibility) and have the appropriate resources and strategies to deal with it (manageability) and feel motivated to face it (meaningfulness). Previous studies on the pandemic point to the important role of SOC on mental health and well-being^(9-11)^.

An integrative review on factors associated with mental health in undergraduate students pointed out that a high SOC score can be a protective factor, capable of influencing coping strategies and reducing psychological distress^(12)^. A Brazilian study showed that students with lower SOC had almost 3 times higher prevalence of using drugs that act on the Central Nervous System, which in turn shows the relationship between SOC and mental disorders^(13)^. Such findings highlight the need for further studies exploring interventions aimed at increasing SOC in promoting mental health.

#### Social coping resource: perceived social support

Social support is a resource widely explored in previous literature^(14-15)^. It is the subjective feeling of having healthy relationships based on support and trust. Such relationships have been shown to be beneficial for health and for successfully coping with stressful situations^(16)^, including in this pandemic^(17-18)^.

Many men, meanwhile, have not received or realized support from those around them during the COVID-19 crisis. A study with men showed that a minority reported receiving social support (37.9%), but among those who did, family support constituted an important coping strategy^(19)^. Thus, it is important to emphasize that the support provided by family members, friends and co-workers as well as by qualified professionals can contribute to emotional control in the face of the perception of threats. A review concluded that it is necessary to expand and strengthen bonds and networks of social support and protection as a way of taking care of the population’s mental health as well as specific groups such as health professionals^(20)^.

#### National coping resources: sense of national coherence and trust in institutions

Sense of national coherence (SONC) was developed by experienced researchers in the field of salutogenesis, who identified the need to develop an instrument that identified the SOC at the national level^(21)^, addressing individuals’ perception about the country’s relevance, management and future as well as its ability to respond to society’s needs. SONC is based on the three SOC components, i.e., the perception of their own nation as comprehensible, manageable and a source of meaning.

A comparative study of four countries identified SONC as an important resource for mental health during the pandemic^(22)^. Trust in institutions was also assessed, as some studies in the early stages of the crisis found that this perception could help people to have better mental health^(23-24)^.

#### Demographic and situational variables

Demographic, social and economic variables have been shown to be important aspects of mental health. Women, in general, have shown to be more likely to develop mental and psychological disorders, including anxiety^(25)^. This pattern continued throughout the COVID-19 pandemic^(26)^. Mental disorders are also often associated with lower income and education^(27)^. Furthermore, it has been reported that income inequality plays a negative role in mental health and well-being^(28)^. Spirituality and religiosity, on the other hand, have been shown to be protective factors during difficult times, being associated with lower levels of worry, fear, sadness^(29)^, and stress^(30)^.

The pandemic has affected people’s lives and well-being in many ways. Fears about COVID-19 added to disease control measures, which completely changed people’s daily lives and income, led to negative psychological effects^(31)^. In this context of crisis, situational factors acquire particular importance. The direct contact with people with COVID-19, as it happens with health students and professionals, seems to have increased anxiety symptom intensity, for instance^(32-33)^.

Former comparative studies tested the model during the outbreak of COVID-19 among participants from 7 European countries and revealed that SOC was strongly associated with mental health in all the samples. Perceived family support and trust in institutions mediated the relationships between SOC and mental health, after controlling for gender, level of exposure to the virus, and level of health and financial risk^(34)^.

The current study tested the model in the unique context of Brazil so that the knowledge acquired can inform future interventions aimed at promoting mental health. In summary, the present study focused on resources that can help people to protect and promote their mental and emotional health, even under severe stressful situations^(35)^.

The study hypotheses:

1. Coping resources will be negatively correlated to anxiety and positively correlated to mental health.
2. SOC, social support, SONC and trust in institutions will predict anxiety and mental health among Brazilians.
3. SOC (personal resource) will be the main predictor of both outcomes.

## OBJECTIVES

To verify the role of SOC, SONC, social support and trust in institutions to predict mental health in Brazilians during the COVID-19 pandemic.

## METHODS

### Ethical aspects

The study complied with national and international ethics guidelines. Prior to data collection, the study was approved by the Research Ethics Committee of *Centro Universitário de Maringá- UniCesumar* and all participants signed the online Informed Consent Form.

### Study period and place

This is a quantitative, cross-sectional study guided by the STrengthening the Reporting of OBservational studies in Epidemiology (STROBE) recommendations.

Data collection was carried out from November 11, 2020 to January 18, 2021 via an online survey on the Qualtrics platform. The invitation letter was widely disseminated on personal, professional and institutional social media (Facebook^®^, Instagram^®^ and LinkedIn^®^) linked to the Brazilian researchers. Moreover, approximately 4,500 distance education students at a private university were invited to participate in the study by email as well as oriented to share the link on their social medias. The dissemination of the study to these students was carried out by the university’s marketing team.

The period of data collection was marked by a considerable increase in the number of cases and deaths from COVID-19 in Brazil. On November 11, 2020, Brazil registered 5,748,375 accumulated cases of and 163,373 accumulated deaths from COVID-19. On January 18, 2021, there were 8,511,770 accumulated cases of and 210,299 accumulated deaths from COVID-19^(36)^.

### Population, sample and eligibility criteria

The research was widely disseminated on social media, and the sample was enriched by undergraduate students, graduate students, masters’ and doctoral degree holders, since a university strongly promoted the study to its students of distance courses through e-mails. The self-reported questionnaires were addressed to Brazilians residing in Brazil and aged 18 or older. Only incomplete questionnaires were eliminated. Thus, 2,601 people accessed the instrument but only 1,630 completed it, excluding 971 participants. The reasons for dropping out were not investigated since no data that could identify the participants were collected. Participants did not receive any financial or other incentives to participate. Participation was voluntary and confidential.

Sample size was based on the Brazilian population aged 19 to 79 years estimated for 2021. Thus, data from the 2010 Brazilian Institute of Geography and Statistics (IBGE - *Instituto Brasileiro de Geografia e Estatística*) census were used, adjusted for population growth from 2010 to 2021 of 10.6%, being the population estimated at 21,119,837 in 2021. The calculation was performed using the OpenEpi software version 3, considering an error of 5% and a significance level of 99%. As a result, the minimum sample size for the research was 664 participants.

### Study protocol

This study is part of a larger project entitled “Corona Research Collaboration”, coordinated by the Ben Gurion University, Israel. It is an initiative of the Society for Theory and Research on Salutogenesis (STARS). STARS was founded in 2017 by the Global Working Group on Salutogenesis of the International Union for Health Promotion and Education (IUHPE). The project brought together several countries around the theme of coping resources and mental health during the COVID-19 pandemic. Originally in English, the study was translated into other languages and applied in several countries such as Israel, Spain, Italy, Netherlands, Austria, Germany, Switzerland, Nigeria, Canada, USA and Brazil.

Instruments previously validated in Brazil were used, when available. Regarding the instruments not validated, such as the social support, trust in relevant institutions and SONC questionnaires as well as questions related to COVID-19, the authors carried out the translation and back-translation, with subsequent assessment and approval by the study’s coordinating team. The next step was approval by the Research Ethics Committee and the insertion of the questionnaires in the Qualtrics platform. Then, the research was disseminated on digital social media, in graduate program research groups as well as via e-mail to distance education undergraduate students.

Through a link, people interested in participating received important information about the purpose and characteristics of the study, ethical issues and inclusion criteria. Those who chose to proceed completed the Informed Consent Form and only then had access to the instruments. The questionnaires addressed mental health through instruments that assessed anxiety and positive mental health. The next chunks analyzed personal (SOC), social (social support) and national (trust in institutions and SONC) coping resources. And finally, sociodemographic, health and financial risk perception data were collected.

### Study instruments

The study instruments comprised structured and self-reported questionnaires. It included instruments previously validated in Brazil such as the generalized anxiety disorder-7, mental health continuumshort form and sense of coherence-13 and others that were back-translated^(37)^ from English to Brazilian Portuguese, such as SONC, social support and trust in relevant institutions and people questionnaires. Questionnaires addressing social support, trust in relevant institutions and people, financial and health risks related to COVID-19 were developed by researchers from the project called “Corona Research Collaboration”, mentioned in more detail in study protocol.

- Generalized anxiety disorder-7 (GAD-7)
  The Brazilian-validated version by Mapi Research Institute^(38)^ was used. Further validity confirmed the GAD-7 scale suitability to be applied to Brazilian adults^(39)^. The GAD-7 is a brief and efficient tool for screening probable cases of generalized anxiety disorder (GAD) consisting of seven items that score from 0 to 3, with the total score consequently ranging from 0 to 21^(40)^. In the current study, the questionnaire’s internal consistency was α= 0.90.
- Mental health continuumshort form (MHC-SF)
  The Brazilian-validated version by Machado and Bandeira^(41)^, a 14-item scale for positive mental health assessment, was used. MHC-SF measures emotional, psychological, and social well-being, on a 6-point Likert scale, ranging from (1) never to (6) every day^(42)^. In the current study, the questionnaire’s internal consistency was α= 0.91.
- Sense of coherence-13 (SOC-13)
  SOC-13, Brazilian-validated version by Spadoti Dantas et al.^(43)^, was used. The instrument developed by Antonovsky^(35)^ consists of 13-items on a 7-point Likert scale that explore participants’ perceptions of the world as comprehensible, meaningful and manageable. In the current study, the questionnaire’s internal consistency was α= 0.86.
- Sense of national coherence (SONC)
  SONC is an 8-items instrument, on a 7-point Likert scale, developed by Mana et al.^(21)^ to assess people’s tendency to perceive the country and the society they belong to as comprehensible, meaningful and manageable. The questionnaire’s internal consistency in the current study was α= 0.81.
- Social support
  A 7-items questionnaire, on a 5-point Likert scale, explored how people felt supported by family, friends, neighborhood, virtual community and other institutions. The questionnaire’s internal consistency in the current study was α= 0.78.
- Trust in relevant institutions and people
  The questionnaire assessed people’s confidence in the media, legal courts, police, presidency, Ministry of Finance, Ministry of Health, Ministry of Education, state and local government, hospitals doctors and health-care workers, on a 5-point Likert scale. The questionnaire’s internal consistency in the current study was α= 0.85.
- Socio-demographic variables
  The questionnaire accessed gender, age, marital status, income, educational level and other demographic information.
- Health and financial risk related to COVID-19
  Participants were asked about personal and family quarantine, personal and family diagnoses, belonging to a risk group and being financially affected by the pandemic.

### Data analysis

Data were analyzed using SPSS Statistics 21. The questionnaire’s internal consistency was calculated, which obtained good values for Cronbach’s alpha (α= > 0.700). A descriptive analysis of the sample’s socioeconomic characteristics, risk factors related to COVID-19 and coping resources was carried out, where variables presented a normal distribution. In relation to group comparisons, t-tests were performed when analyzing two groups, and a one-way ANOVA with Tukey´s test when comparing 3 or more groups. Correlation between mental health, anxiety and coping resources were performed using a Pearson’s correlation coefficient. Multiple linear regression analysis assessed the extent to which the set of resources (independent variables) were able to predict mental health and anxiety (dependent variables), and also examined which resources in particular were predictors of the outcomes. Demographic variables such as gender, age, income and marital status were controlled in the model. Most importantly, there were no multicollinearity issues between independent variables (SOC, SONC, social support and trust in relevant institutions/people), since all variance inflation factors were below 3.

## RESULTS

The study comprised 1,630 Brazilians, of which 71.1% were women. Participants’ age ranged from 18 to 76 years (M=36.2, SD= 10.59). Overall, 58.7% of them reported to be married/common law marriage and 51.1% to have one or more children under the age of 18 in the household. According to participants, 52.8%% had up to 12 years of education (corresponding to high school) and 47.2% were undergraduate students, or already had a master’s or doctoral degree. Furthermore, 46.2% received a monthly amount of up to 2 minimum wages, which represents a scarce financial resource. About 89% reported having a religion, and 28.3% identified themselves politically more as right wing, and 13.7, more as left wing. The study reached a geographic diversity, with participants from all regions of Brazil, although in different proportions, as it was a convenience sampling. Full sociodemographic characteristics are depicted in Table 1.

**Table 1:** Sociodemographic and COVID-19-related characteristics of participants (N = 1,630)

Variables	Frequency (n)	Percentage (%)
Gender		
Male	471	28.9
Female	1159	71.1
Marital status		
Single	501	30.7
Married/common law marriege	956	58.7
Divorced	104	6.4
Widowed	15	0.9
Other	54	3.3
Children under 18 years		
Yes	833	51.1
No	797	48.9
Educational level		
High school or lower	862	52.8
Undergraduate student or higher	768	47.2
Income		
≤ 2 minimum wages	752	46.2
> 2 minimum wages	812	49.8
Preferred not to answer	66	4
Religion		
Catholic	659	40.4
Evangelic	602	36.9
Others	196	12.0
Without religion	173	10.7
Political spectrum		
Right wing	364	22.3
Right-center	98	6
Left wing	145	8.9
Left-center	79	4.8
Not sure or preferred not to answer	944	57.9
Brazilian regions		
South	536	32.9
Southeast	703	43.1
Midwest	103	6.3
North	99	6.1
Northeast	189	11.6
Belong to a risk group		
No	1326	81.3
Yes	304	18.7
Personal quarantine		
No	1000	61.3
Yes	630	38.7
Family quarantine		
No	810	49.7
Yes	820	50.3
Personal diagnosis		
No	1482	90.9
Yes	148	9.1
Family diagnosis		
No	1065	65.3
Yes	565	34.7
Financial risk		
Little or not affected	807	49.5
Very or extremely affected	823	50.5

Some questions addressed the risks associated with the COVID-19 pandemic and confirmed what was happening in most of the Brazilian territory during data collection. Most participants did not belong to a risk group (81.3%) and had not been in quarantine (61.3%). The vast majority had not been diagnosed with COVID-19 (90.9%), and the majority had no diagnosis in the family (65.3%). Additionally, a perception of great economic impact was noticed, with 50.5% believing that they would be very or extremely financially affected. More details are presented in Table 1.

### Descriptive statistics

In terms of mental health, anxiety and coping resources, participants scored on average 4.2 on MHC-SF (SD= 1), 10.1 on GAD-7 (SD= 5.73), 4.4 on SOC-13 (SD=1.19), 3.4 on SONC (SD=1.26), 3.1 on social support (SD=0.85) and 2.7 on trust in relevant institution questionnaire (SD=0.77).

### Group comparisons

Table 2 shows the means of anxiety and mental health according to sociodemographic and COVID-19 related variables. Comparing the groups, significant lower averages of mental health and higher averages of anxiety were found among females, adults aged 18 to 29 years, single, and those with lower income, without religion, who were left-wing, in quarantine or who had a family member in quarantine, who had a family member diagnosed with COVID-19 and who felt they would be more seriously financially affected by the pandemic. Having children in the household was associated with better mental health but higher levels of anxiety.

**Table 2:** Means and standard deviation of anxiety and mental health according to sociodemographic and COVID-19 related variables

Variables	GAD-7 (Anxiety)			MHC-SF (Mental health)		
	M	SD	*p value*	M	SD	*p* *value*
Gender						
Female	10.50	5.77	<0.001	4.09	1.01	<0.001
Male	9.03	5.52		4.34	0.97	
Age						
18-29	11.67	5.58	<0.001	3.73	1.03	<0.001
30-39	9.97	5.81		4.15	0.97	
40-49	9.33	5.58		4.43	0.88	
50-59	7.98	5.32		4.65	0.82	
>=60	8.75	4.97		4.81	0.92	
Marital status						
Single	11.10	5.60	<0.001	3.81	1.07	<0.001
Married	9.42	5.67		4.33	0.93	
Divorced	10.79	5.97		4.22	0.98	
Widowed	8.80	6.90		4.60	0.79	
Children in household						
No	9.74	5.77	0.021	4.10	1.04	0.015
Yes	10.40	5.70		4.22	0.96	
Educational level						
High school or lower	10.36	5.68	0.031	4.12	1.00	0.180
Undergraduate student or higher	9.74	5.79		4.20	1.00	
Income						
≤ 2 minimum wages	10.69	5.74	<0.001	4.08	1.02	0.005
> 2 minimum wages	9.53	5.68		4.22	0.98	
Religion						
No	11.13	6.22	0.011	3.61	1.08	<0.001
Yes	9.95	5.67		4.23	0.97	
Political spectrum						
Right wing	8.87	5.65	<0.001	4.43	0.98	<0.001
Center-right	8.15	5.25		4.23	0.98	
Left wing	11.37	5.76		3.84	1.11	
Center-left	9.96	5.27		3.91	1.09	
Brazilian regions						
South	10.20	5.73	0.361	4.10	1.00	0.081
Southeast	10.22	5.84		4.15	1.01	
Midwest	9.62	6.02		4.32	0.97	
North	9.12	5.37		4.36	0.95	
Northeast	9.83	5.43		4.19	1.05	
Belong to a risk group						
No	9.91	5.68	0.016	4.16	1.01	0.996
Yes	10.79	5.96		4.16	0.98	
Personal quarantine						
No	9.80	5.80	0.014	4.23	0.99	0.001
Yes	10.51	5.62		4.06	1.02	
Family quarantine						
No	9.52	5.67	<0.001	4.26	0.96	<0.001
Yes	10.62	5.76		4.06	1.04	
Personal diagnosis						
No	10.06	5.75	0.685	4.15	1.01	0.058
Yes	10.26	5.60		4.31	0.98	
Family diagnosis						
No	9.79	5.75	0.006	4.20	0.99	0.041
Yes	10.61	5.68		4.09	1.01	
Financial risk						
Little or not affected	8.78	5.49	<0.001	4.30	0.94	<0.001
Very or extremely affected	11.34	5.69		4.02	1.05	

*GAD-7 - Generalized anxiety disorder-7; MHC-SF - Mental health continuumshort form.*

Those who reported having studied up to high school had higher levels of anxiety than those who were undergraduate or had already completed a university, master’s or doctoral course as well as belonging to a risk group that was also associated with increased anxiety. Living in different regions of the country and being diagnosed with COVID-19 were not associated with the outcomes, as seen in Table 2.

### Correlations

Correlations between anxiety, mental health and coping resources are shown in Table 3. As predicted, all correlations between mental health (MHC-SF) and the resources (SOC-13, social support, trust in relevant institutions and SONC) were significant (p<0.001) and positive. So, positive mental health was directly correlated to SOC-13 (0.67), SONC (0.42), social support (0.41) and trust in relevant institutions (0.27). In other words, the higher the participants’ mental health, the greater the SOC, the SONC, the perceived social support and the trust in relevant institutions.

**Table 3:** Correlations between the mental health, anxiety and other instruments

	MHC-SF	GAD-7
Sense of coherence	0.67^**^	-0.62^**^
Social support	0.41^**^	-0.21^**^
Trust in Institutions	0.27^**^	-0,10^**^
Sense of national coherence	0.42^**^	-0,25^**^

MHC-SF - Mental health continuumshort form; GAD-7 - Generalized anxiety disorder-7;

** p<0.001.

Table 3 reveals that the correlations between anxiety (GAD-7) and the resources were also significant (p<0.001) and, unsurprisingly, negative. In this way, the anxiety level was inversely correlated with SOC-13(-0.62), SONC (-0.25), social support (-0.21) and trust in relevant institutions (-0.10). This means that a stronger SOC, SONC, greater social support and trust in relevant institutions are associated with lower levels of anxiety. Thus, the first hypothesis was confirmed.

Although significant, the correlation between mental health and trust in institutions was weak, and the strongest correlation was between mental health and SOC. With regard to anxiety, the correlations were weak, except for the correlation with SOC, which was moderate, as can be seen in Table 3.

### Linear Regression

Demographic variables such as gender, age, marital status and income were controlled in the model. Table 4 shows the linear regression analysis for anxiety. The coefficient of determination, R^2^ = 0.384, [F (8, 1629) = 128.16, p<0.001)], reveals that the combination of predictors explained 38.4% of the variance in anxiety scores. Furthermore, SOC was the only significant predictor for anxiety.

**Table 4:** Linear regression model using sense of coherence, sense of national coherence, trust in institutions and social support as predictor variables for anxiety

Predictor variables	b	95% CI		β	*p* *value*
		LL	UL		
Gender	0.43	-0.07	0.92	0.03	0.092
Age	0.02	-0.22	0.26	0.00	0.857
Marital status	0.25	-0.03	0.52	0.04	0.078
Income	-0.13	-0.34	0.08	-0.02	0.220
Sense of coherence	-2.94	-3.16	-2.73	-0.61	<0.001
Social support	-0.13	-0.43	0.17	-0.02	0.347
Trust in institutions	0.30	-0.04	0.63	0.04	0.077
Sense of national coherence	-0.09	-0.30	0.11	-0.02	0.370

*b-unstandardized regression coefficient, LL- lower limit, UL- upper limit of 95% confidence interval (CI).*

Table 5 presents the linear regression analysis for mental health. The coefficient of determination, R^2^ = 0.513, [F (8, 1629) = 215.88, p <0.001], shows that the set of predictor variables explained 51.3% of the variance in mental health scores, pointing out that the model represented an important predictor for the outcome. It is notable that mental health was significantly predicted by SOC, social support and SONC, however not by trust in institutions.

**Table 5:** Linear regression model using sense of coherence, sense of national coherence, trust in institutions and social support as predictor variables for mental health

Predictor variables	b	95% CI		β	*p* *value*
		LL	UL		
Gender	-0.08	-0.15	0.00	-0.03	0.050
Age	0.07	0.03	0.11	0.07	<0.001
Marital status	0.06	0.01	0.10	0.05	0.008
Income	-0.03	-0.06	0.00	-0.03	0.054
Sense of coherence	0.44	0.40	0.47	0.52	<0.001
Social support	0.18	0.13	0.22	0.15	<0.001
Trust in institutions	0.04	-0.01	0.10	0.03	0.095
Sense of national coherence	0.13	0.10	0.16	0.16	<0.001

*b-unstandardized regression coefficient, LL- lower limit, UL- upper limit of 95% confidence interval (CI).*

## DISCUSSION

The COVID-19 pandemic was widely analyzed from a pathogenic perspective. The current study, in contrast, employed a salutogenic approach and asked: what are the factors that promote mental health in the difficult situation of COVID-19 in Brazil? We were particularly interested in understanding the role of personal (SOC), social (social support) and national (SONC and trust in institutions) resources in explaining generalized anxiety and positive mental health during the COVID-19 pandemic.

The mental health scores in the Brazilian sample were average. This finding is consistent with former studies reported from Spain, Israel, Italy and the Netherlands^(22)^, indicating that positive mental health is possibly a more stable measure. On the other hand, the level of general anxiety was high, compared to data found in other European countries^(34)^. The high level of anxiety may reflect the stressful period of data collection, when the number of cases and deaths in Brazil increased significantly^(36)^. But it is very likely that it reflects the pre-pandemic anxiety condition. As mentioned earlier, a publication by the World Health Organization in 2017^(2)^ already warned that Brazilians had the highest prevalence of anxiety among all populations. However, our findings revealed that several social groups were more vulnerable to anxiety compared to the others. Women had more anxiety and less mental health compared to men. This direction is a common finding across studies^(26,44)^. Our results also suggest that, during the pandemic, mental health/anxiety was worse among younger people, with lower incomes (lower financial stability), singles (who were probably lonelier during social isolation), those who felt less supported and represented (socially and politically), those who were truly isolated and those who felt most threatened (in relation to health and finances).

Having a religion was associated with better levels of anxiety and mental health. It seems that spirituality and religiosity during the pandemic was a protective factor for health^(29)^, which can be explained by the strengthening of bonds, social cohesion and support, sense of purpose and other possible benefits^(45)^. In addition to this, having children in the household had different results: positive for mental health and negative for anxiety, which makes us reflect on the distinction between the two variables and their determinants. We assume that children’s demands avoided feelings of loneliness. At the same time, many parents found it difficult to manage their personal and professional lives with the care and education of their children^(46)^, which may be responsible for anxiety symptoms.

As for the relationships between coping resources and mental health and anxiety, as we hypothesized, SOC was the main predictor of both reactions in Brazilians during the pandemic. From the WHO perspective, some factors are considered health assets, because they protect health from the damage caused by daily stress and actively promote health, such as resilience and social cohesion^(47)^. In this study, the highest or strongest SOC was the most important health asset. This finding confirmed former studies^(34)^. A prospective study demonstrated that people with the strongest SOC before the pandemic had greater stability in psychopathological symptoms during the crisis^(11)^, while Barni et al.^(9)^ confirmed the role of SOC as a coping resource linked to psychological well-being.

Perceived social support was also a predictor of mental health. Former studies revealed that social support plays an important role in mental health^(14-15)^. In other words, perceiving oneself supported by family, friends, community and the public institutions helps people to maintain mental health, particularly in critical moments such as the pandemic, full of new situations and major challenges that include social isolation, fear of falling ill, stigma, limited information and supplies, and financial uncertainties^(31)^.

Regarding the national coping resources, as expected, SONC was related to mental health. SONC was also a predictor of mental health in Israeli, Dutch and Italian samples, but not in the Spanish during the COVID-19 pandemic^(22)^. However, unexpectedly, trust in governmental institutions was not related to anxiety or mental health. As we tested in the same instrument trust in the media, political institutions, hospital and health professionals, it is possible that each institution had a different weight on reactions^(48)^, and this should be tested in future studies.

The regression analysis revealed that the personal, social and national resources explained 38% of the variation in anxiety and 51% of the variation in mental health. It is proposed that health promotion interventions that strengthen SOC, SONC and consequently resilience could help people to deal with the challenges imposed by the pandemic and feel more prepared to face future crises.

Health promotion actions and more effective communication are essential for empowering communities, in order to make individuals more committed to their own health, the health of others and the environment in which they live. In times of crisis, informative materials are essential, but excess information also needs to be reviewed, as it was responsible for increasing fear and anxiety^(49)^. Furthermore, access to information is not enough to change habits and promote health. Strategies that consider the variables related to following the guidelines are necessary, because only this condition is linked to the reduction of anxiety symptoms^(50)^.

Empowerment would be linked to greater understanding of the events, active participation, perception that they have the necessary internal and external resources, and to the understanding that it is worth mobilizing all available resources to face the challenges. Strategies that facilitate social support are also needed, especially in situations that make people more vulnerable, such as social isolation, health crises and financial threat. A study of men demonstrated that social support moderated the positive relationship between intolerance of uncertainty and mental disorders. It is therefore suggested that social support is a protective factor for mental health and, consequently, a promising intervention resource^(51)^.

### Study limitations

The sample characteristics prevent the generalization of results. The sample consisted predominantly of women and highly educated people, with a large number of undergraduate students and professionals, i.e., there was an imbalance in terms of gender and educational level associated with the ways in which the study was disseminated and the convenience sample. However, using social medias to carry out the online research, with self-report instruments, was the best means found to continue advancing on important issues the in light of the social restrictions generated by the pandemic, within the available resources. Although recognizing the selection bias resulting from the methodology, it allowed us to reach a large sample, including people from other regions of Brazil.

Furthermore, it is important to highlight that some study instruments, such as the questionnaires on social support, trust in relevant institutions and SONC, were not previously adapted and validated in Brazil. While translation and back-translation were carried out at the highest standards, they are not a substitute for cross-cultural adaptation.

Another limitation of this study is its cross-sectional design, which is not ideal for showing causality. In this regard, we believe that longitudinal studies as well as qualitative approaches would be useful for a better understanding of the relationships between coping resources, anxiety and mental health.

### Contributions to mental health promotion

The manuscript contributes to knowledge in the field of health promotion by focusing on salutogenic factors (coping resources or health assets) related to better mental health of Brazilian adults in a period of crisis. More than ever, the Brazilian population can benefit from interdisciplinary strategies to promote mental health. And interventions that consider the dimensions of SOC (understandability, management and significance) as well as social support have the potential to stand out in this context.

## CONCLUSIONS

Our findings suggest that SOC was an important predictor of anxiety and mental health as well as attributing a significant role to personal (SOC), social (social support) and national (SONC) resources to the Brazilian population’s mental health during the pandemic. Such findings should be considered with caution, as the studied sample represents a partial picture of the Brazilian population’s reality. Moreover, other important resources may be acting together to explain the variance in levels of anxiety and mental health.

The strengths of the study included sample size and composition, which included people from all Brazilian regions. Furthermore, being part of an international collaboration allowed relevant comparisons while analyzing the unique context of Brazil. A possible recommendation in the light of the results is investment in multiple interventions aimed at empowering individuals and communities. In this process, we attributed an important role to the strengthening of the sense of personal and national coherence associated with social support, which proved to be effective resources for mental health and anxiety reduction during health crises.

## Data Availability

https://doi.org/10.48331/scielodata.QTPK7E

## References

[1] Feter N, Caputo EL, Doring IR (2021). Sharp increase in depression and anxiety among Brazilian adults during the COVID-19 pandemic: findings from the PAMPA cohort. Public Health.

[2] World Health Organization (WHO) (2017). Depression and other common mental disorders: global health estimates [Internet].

[3] Morin CM, Bjorvatn B, Chung F. (2021). Insomnia, anxiety, and depression during the COVID-19 pandemic: an international collaborative study. Sleep Med.

[4] Grillon C, Duncko R, Covington MF. (2007). Acute stress potentiates anxiety in humans. Biol Psychiatry.

[5] World Health Organization (WHO) (2004). Promoting mental health: concepts, emerging evidence, practice (Summary Report) [Internet].

[6] Mittelmark MB, Bauer GF., Mittelmark MB, Sagy S, Eriksson M (2017). The Handbook of Salutogenesis.

[7] Vindegaard N, Benros ME. (2020). COVID-19 pandemic and mental health consequences: systematic review of the current evidence. Brain Behav Immun.

[8] Antonovsky A. (1996). The salutogenic model as a theory to guide health promotion. Health Promot Int.

[9] Barni D, Danione F, Canzi E. (2020). Facing the COVID-19 pandemic: the role of sense of coherence. Front Psychol.

[10] Généreux M, Schluter PJ, Hung KK (2020). One virus, four continents, eight countries: an interdisciplinary and international study on the psychosocial impacts of the COVID-19 pandemic among adults. Int J Environ Res Public Health.

[11] Schäfer SK, Sopp MR, Schanz CG. (2020). Impact of COVID-19 on public mental health and the buffering effect of a sense of coherence. Psychother Psychosom.

[12] Graner KM, Cerqueira ATAR. (2019). Revisão integrativa: sofrimento psíquico em estudantes universitários e fatores associados. Ciênc Saúde Colet.

[13] Abreu VSM, Teles DO, Rodrigues HBV, Pires JM, Soares PRAL, Aquino OS (2022). Risk factors for Central Nervous System drug use among nursing students. Rev Bras Enferm.

[14] Srensen T, Klungsyr O, Kleiner R. (2011). Social support and sense of coherence: independent, shared and interaction relationships with life stress and mental health. Int J Mental Health Promot.

[15] Sehmi R, Maughan B, Matthews T, Arseneault L. (2020). No man is an island: social resources, stress and mental health at mid-life. Brit J Psychiatr.

[16] Taylor SE., Friedman HS (2012). The Oxford Handbook of Health Psychology.

[17] Kimhi S, Marciano H, Eshel Y. (2020). Recovery from the COVID-19 pandemic: distress and resilience. Int J Disaster Risk Reduct.

[18] Szkody E, Stearns M, Stanhope L, McKinney C. (2021). Stress-buffering role of social support during COVID-19. Fam Process.

[19] Sousa AR, Teixeira JRB, Mota TN, Santana TS, Santos SD, Merces MC. (2021). Coping strategies, concerns, and habits of Brazilian men in the COVID-19 context. Rev Bras Enferm.

[20] Moreira WC, Sousa KHJF, Sousa AR, Santana TS, Zeitoune RCG, Nóbrega MPSS. (2021). Mental health interventions implemented in the COVID-19 pandemic: what is the evidence?. Rev Bras Enferm.

[21] Mana A, Srour A, Sagy S. (2019). A sense of national coherence and openness to the “other’s” collective narrative: the case of the Israeli- Palestinian conflict. Peace and Conflict: J Peace Psychol.

[22] Mana A, Super S, Sardu C. (2021). Individual, social and national coping resources and their relationships with mental health and anxiety: a comparative study in Israel, Italy, Spain and the Netherlands during the Coronavirus pandemic. Glob Health Promot.

[23] Jetten J, Reicher SD, Haslam SA (2020). Together apart: the psychology of COVID-19.

[24] Žižek S. (2020). New.

[25] Russo NF, Tartaro J., Denmark FL, Paludi MA (2008). Psychology of women: a handbook of issues and theories.

[26] Connor J, Madhavan S, Mokashi M. (2020). Health risks and outcomes that disproportionately affect women during the Covid-19 pandemic: a review. Soc Sci Med.

[27] Goularte JF, Serafim SD, Colombo R, Hogg B, Caldieraro MA, Rosa AR. (2021). COVID-19 and mental health in Brazil: psychiatric symptoms in the general population. J Psychiatr Res.

[28] Ribeiro WS, Bauer A, Andrade MCR, York-Smith M, Pan PM, Pingani L (2017). Income inequality and mental illness-related morbidity and resilience: a systematic review and meta-analysis. Lancet Psychiatry.

[29] Lucchetti G, Góes LG, Amaral SG, Gonzalez-Manso MA. (2021). Spirituality, religiosity and the mental health consequences of social isolation during Covid-19 pandemic. Int J Soc Psychiatry.

[30] Ting RS, Aw Yong YY, Tan MM, Yap CH. (2021). Cultural responses to Covid-19 pandemic: religions, illness perception, and perceived stress. Front Psychol.

[31] Brooks SK, Webster RK, Smith LE, Woodland FC. (2020). The psychological impact of quarantine and how to reduce it: rapid review of the evidence. Lancet.

[32] Dantas ESO, Araújo JD, Silva GWS, Silveira MYM, Dantas MNP, Meira KC. (2021). Factors associated with anxiety in multiprofessional health care residents during the COVID-19 pandemic. Rev Bras Enferm.

[33] Dal’Bosco EB, Floriano LSM, Skupien SV, Arcaro G, Martins AR, Anselmo ACC. (2020). Mental health of nursing in coping with COVID-19 at a regional university hospital. Rev Bras Enferm.

[34] Mana A, Bauer GF, Meier-Magistretti C, Sardu C, Juvinyà-Canal D, Hardy LJ (2021). Order out of chaos: sense of coherence and the mediating role of coping resources in explaining mental health during COVID-19 in 7 countries. SSM Ment Health.

[35] Antonovsky A. (1987). Unraveling the mystery of health: how people manage stress and stay well.

[36] Ministério da Saúde (BR) (2021). Painel coronavírus [Internet].

[37] Tyupa S. (2011). A theoretical framework for back-translation as a quality assessment tool. New Voices Transl Stud [Internet].

[38] Mapi Research Institute (2006). Certificate of linguistic validation certificate: General Anxiety Disorder-7 (GAD-7).

[39] Moreno AL, DeSousa DA, Souza AMFLP. (2016). Factor structure, reliability, and item parameters of the brazilian-portuguese version of the GAD-7 questionnaire. Temas Psicol.

[40] Spitzer RL, Kroenke K, Williams JBW, Lowe B. (2006). A brief measure for assessing generalized anxiety disorder: the GAD-7. Arch Intern Med.

[41] Machado WL, Bandeira DR. (2015). Positive mental health scale: validation of the Mental Health Continuum - Short Form. Psico-USF.

[42] Lamers SMA, Westerhof GJ, Bohlmeijer ET, ten Klooster PM, Keyes CLM (2011). Evaluating the psychometric properties of the Mental Health Continuum-Short Form (MHC-SF). J Clin Psychol.

[43] Spadoti Dantas RA, Silva FS, Ciol MA. (2014). Psychometric properties of the Brazilian Portuguese versions of the 29- and 13-item scales of the Antonovsky’s Sense of Coherence (SOC-29 and SOC-13) evaluated in Brazilian cardiac patients. J Clin Nurs.

[44] Chekole YA, Abate SM. (2021). Global prevalence and determinants of mental health disorders during the COVID-19 pandemic: a systematic review and meta-analysis. Ann Med Surg (Lond).

[45] Eckersley RM. (2007). Culture, spirituality, religion and health: looking at the big picture. Med J Australia.

[46] Spinelli M, Lionetti F, Pastore M, Fasolo M. (2020). Parents’ stress and children’s psychological problems in families facing the COVID-19 outbreak in Italy. Front Psychol.

[47] Morgan A, Ziglio E. (2007). Revitalising the evidence base for public health: an assets model. Promot Educ.

[48] Russo GA, Azzi RG, Faveri C. (2018). Confiança nas instituições políticas: diferenças e interdependência nas opiniões de jovens e população brasileira. Opinião Pública.

[49] Delgado CE, Silva EA, Castro EAB, Carbogim FC, Püschel VAA, Cavalcante RB. (2021). COVID-19 infodemic and adult and elderly mental health: a scoping review. Rev Esc Enferm USP.

[50] Pilz LK, Pereira NSC, Francisco AP, Carissimi A, Constantino DB, Caus LB (2022). Effective recommendations towards healthy routines to preserve mental health during the COVID-19 pandemic. Braz J Psychiatry.

[51] Palma EMS, Sousa AR, Moraes FA, Teixeira JRB, Sousa ÁFL. (2022). Men’s mental health in the COVID-19 pandemic: the role of intolerance of uncertainty and social support. Paidéia (Ribeirão Preto).

